# Rapid SARS-CoV-2 Detection Using Electrochemical Immunosensor

**DOI:** 10.3390/s21020390

**Published:** 2021-01-08

**Authors:** Biljana Mojsoska, Sylvester Larsen, Dorte Aalund Olsen, Jonna Skov Madsen, Ivan Brandslund, Fatima AlZahra’a Alatraktchi

**Affiliations:** 1 Department of Science and Environment, Roskilde University, Universitetsvej 1, 4000 Roskilde, Denmark; Biljana@ruc.dk (B.M.); syla@ruc.dk (S.L.); 2 Department of Biochemistry and Immunology, Lillebaelt Hospital, University Hospital of Southern Denmark, 7100 Vejle, Denmark; Dorte.Aalund.Olsen@rsyd.dk (D.A.O.); Jonna.Skov.Madsen@rsyd.dk (J.S.M.); Ivan.Brandslund@rsyd.dk (I.B.); 3 Department of Regional Health Research, Faculty of Health Sciences, University of Southern Denmark, 5230 Odense, Denmark

**Keywords:** SARS-CoV-2, COVID-19, 2019-nCoV, biosensor, immunosensor, electrochemical sensing, voltammetry, spike protein, diagnostics, corona

## Abstract

The outbreak of the coronavirus disease (COVID-19) pandemic caused by the novel coronavirus (SARS-CoV-2) has been declared an international public health crisis. It is essential to develop diagnostic tests that can quickly identify infected individuals to limit the spread of the virus and assign treatment options. Herein, we report a proof-of-concept label-free electrochemical immunoassay for the rapid detection of SARS-CoV-2 virus via the spike surface protein. The assay consists of a graphene working electrode functionalized with anti-spike antibodies. The concept of the immunosensor is to detect the signal perturbation obtained from ferri/ferrocyanide measurements after binding of the antigen during 45 min of incubation with a sample. The absolute change in the [Fe(CN)_6_]_3_−/^4−^ current upon increasing antigen concentrations on the immunosensor surface was used to determine the detection range of the spike protein. The sensor was able to detect a specific signal above 260 nM (20 µg/mL) of subunit 1 of recombinant spike protein. Additionally, it was able to detect SARS-CoV-2 at a concentration of 5.5 × 10^5^ PFU/mL, which is within the physiologically relevant concentration range. The novel immunosensor has a significantly faster analysis time than the standard qPCR and is operated by a portable device which can enable on-site diagnosis of infection.

## 1. Introduction

Coronavirus disease 2019 (COVID-19) which is caused by the severe acute respiratory syndrome coronavirus 2 (SARS-CoV-2) was declared a global pandemic in early 2020 and has infected more than 82 million and killed more than 1.8 million people worldwide to date [1,2]. The strategy of many countries to combat the spread of the virus relies on fast identification of infected people and their close contacts. This can result in faster isolation of the infected individuals and a lower transmission or spread of the virus among the population.

The current global standard testing strategy, as recommended by the World Health Organization (WHO, Geneva, Switzerland), for the detection of SARS-CoV-2 virus targets viral RNA using polymerase chain reaction (PCR) [3,4]. The success of this strategy depends on rapid testing; however, since PCR is often carried out at centralized laboratories, test facility bottlenecks can be created, both by transport and batch analysis, extending the time from test to answer. As highlighted by the World Health Organization and the European Commission, one of the main COVID-19 management strategies is related to rapid and accurate analysis including antigen tests [5,6]. The development of supplementary on-site point-of-care (POC) rapid tests could help alleviate the bottlenecks of central testing and open up opportunities to hold larger gatherings by the prevention of super-spreader events. The importance of rapid testing for the prevention of new cases has been discussed widely [7,8]. At the time of publishing of this paper, more than 140 commercial Sars-CoV-2 tests have received the status of Emergency Use Authorization (EUA) for diagnostic use by the FDA [9]. However, the vast majority of these are RT-PCR-based and detect the viral RNA, with only a handful of antigen tests being approved. Most antigen tests target the nucleocapsid protein, which is one of the four major structural proteins of the SARS-CoV-2 virus [10,11]. However, the Spike (S) protein, which is another structural protein, has less sequence homology with the previous SARS-CoV and MERS viruses, and might thus be a more specific antigen target.

Electrochemical biosensors provide a promising approach to rapid medical diagnostics of infection-biomarkers that may lead to more timely and effective decision-making [12,13,14,15]. Furthermore, potentiostats are already available in miniaturized formats, enabling the possibility of on-the-spot or point-of-care applications. Electrochemical biosensors have previously been used to detect viral antigens from several pathogenic viruses such as Hepatitis, Dengue, Rabies, and Zika [16,17,18]. The ongoing COVID-19 pandemic has led to an increase in biosensor research aiming to identify and quantify SARS-CoV-2 specific antigens [19,20,21,22]. Seo et al. have developed a Field-Effect Transistor-Based (FET) biosensor by coating a graphene surface with a monoclonal antibody against the SARS-CoV-2 Spike protein, obtaining a detection limit of 1 fg/mL in PBS [23]. Mahari et al. have also shown a proof-of-concept of a similar screen-printed electrode (SPE) biosensor against the Spike antigen, as well as a homemade potentiostat for reading the SPE with a limit of detection of 10 fM in saliva within 30 s [24]. The few current EUA-approved nucleocapsid protein antigen tests can detect approximately 115–100 PFU/mL of SARS-CoV-2 virus. In comparison, commercial PCR-based methods have a sensitivity of approximately 1200 viral genome copies/mL [25].

In this work, we present a proof-of-concept novel electrochemical biosensor for rapid detection of SARS-CoV-2 Spike antigen, using SPE functionalized with a monoclonal anti-spike antibody. The sensor consists of a printed graphene layer functionalized with a 1-pyrene butyric acid N-hydroxysuccinimide ester (PBASE) linker bound to a monoclonal anti-spike antibody. The time for incubation and testing using the herein presented immunosensor is 45 min, which is significantly lower than what is possible by PCR. The biosensor can specifically detect the Spike protein with the lowest detected concentration of 260 nM (20 µg/mL) using a 45 min incubation time and a SARS-CoV-2 concentration of 5.5 × 10^5^ PFU/mL. The immunosensor developed here is a proof-of-concept that, despite its lower sensitivity compared to PCR, might still be valuable in a setting where quick decision-making is necessary.

## 2. Materials and Methods

### 2.1. SARS CoV-2 Automated Single Molecule Array Assay

The measurement of SARS CoV-2 S-protein was performed on an automated single-molecule array (Simoa) HD-1 Analyzer platform (Quanterix©). In short, the Spike-protein capture antibody (#40150-D006, Sino Biological Beijing, China) was covalently attached by standard carbodiimide coupling chemistry to carboxylated paramagnetic beads (Quanterix) using 0.2 mg/mL antibody and 0.3 mg/mL EDC (#77149, Thermo Fisher Scientific, Waltham, MA, USA). The spike protein detection antibody (#40591-MM43, Sino Biological) was biotinylated using a 40:1 molar ratio of biotin (#A39259, Thermo Fisher Scientific) to antibody. Recombinant Spike subunit 1 protein was applied as a calibrator (#40592-V08B, Sino Biological). 22E6 PFU/mL recombinant SARS CoV-2 virus (Linköping University, Linköping, Sweden) was diluted in PBS with 1% BSA, followed by a two-fold dilution in lysis buffer (100 mM Tris-HCl, 800 mM NaCl, pH 9.0, 1% Triton x-100, 1% BSA).

The method was developed as a single-plex assay using a two-step protocol. The following reagents were prepared beforehand: capture beads (1/3) were mixed with helper beads (2/3) in bead diluent buffer (Quanterix, Billerica, MA, USA) and diluted to a final concentration of 2.0 × 107 beads/mL. The biotinylated detection antibody was diluted in sample/detection diluent (Quanterix) to a final concentration of 0.4 ug/mL. The streptavidin-beta-galactosidase (SβG) was diluted in SβG diluent (Quanterix) to 50 pM. Upon loading the reagents and consumables, eight calibrators were prepared, starting from 300 pg/mL S-protein and titrated using a three-fold dilution. Calibrators, samples and internal assay control materials were loaded to the instrument in a 96-well microtiter plate.

The instrument performed the following steps: (1) A total of 25 µL of capture beads were pipetted into a cuvette together with 100 µl of sample, control or calibrator and 50 µL of biotinylated detection antibody. (2) Incubation was performed for 30 min. (3) The beads were magnetically separated and washed. (4) Then, 100 µL of SβG was added to the cuvette by the instrument. (5) Incubation was performed for 5 min. (6) After washing the beads, RGP substrate was added. (7) Finally, the beads were loaded on to the Simoa disc containing an array of 216,000 micro-wells and sealed with oil. The average number of enzymes per bead was used as a readout. Using four parameters logistic fitting, the protein concentration was interpolated from the calibrator curve [26].

### 2.2. ELISA Preparations

Enzyme-linked immunosorbent assay (ELISA) was performed by coating a 96-well Nunc Maxisorp plate (Thermo Fisher, #44-2404) with 100 µL per well of a 2× dilution series of SARS-CoV-2 Spike S1-His protein (Sino biological, #40591-V08B1) for 16 h at 4 °C. The wells were washed three times with 200 µL PBST (137 mM NaCl, 2.7 mM KCl, 10 mM Na_2_HPO_4_, 1.8 mM KH_2_PO_4_, and 0.05% Tween 20, pH 7.4). The wells were blocked by incubating for 1 h with 100 µL PBST containing 2% Bovine Serum Albumin (BSA) and washed three times. A total of 100 µL of 1:10,000 primary anti-spike antibodies (#45150-D003 and #45150-D001, Sino biological) was added to each well and incubated for 1 h at 37 °C and washed four times. 100 µL of 1:10,000 Goat anti-human-IgG-HRP (#A18817, Thermo Fisher) conjugated antibody was added and incubated for 30 min at room temperature, and wells were washed four times. A total of 100 µL TMB Plus2 (#4395, Kementec, Taastrup, Denmark) HRP substrate was added, and the plate was incubated in the dark for 25 min. The reaction was stopped by the addition of 100 µL 0.2 M H_2_SO_4_ and 450 nm (specific signal) and 620 nm (background) absorbance were read in a standard plate reader after 5 min.

### 2.3. Functionalization of Graphene Electrochemical Sensors

Graphene sensors (#GPH381-2, PreDiagnose, Karlslunde, Denmark) was functionalized by pipetting 50 µL 2 mM 1-Pyrenebutyric acid N-hydroxysuccinimide ester (PBASE) (#114932-60-4, Sigma-Aldrich, St. Louis, MO, USA) dissolved in 1:3 *v/v* of dimethylformamide (68-12-2, Sigma-Aldrich) to methanol (#67-56-1, Sigma-Aldrich) onto the sensor to cover the working electrode. Sensors were covered to avoid evaporation and incubated for 2 h at room temperature. The sensors were then washed with methanol and dried with nitrogen. The anti-spike antibody (#45150-D003, Sino biological) was diluted 100x in PBS to approximately 22 µg/mL, and 10 µL was added to the working electrode and incubated for 2 h at 37 °C and 300 rpm shaking. After washing and drying the sensors with PBS, they were blocked by the addition of 50 µL PBST (0.05% Tween-20) containing 1% BSA to the working electrode, and sensors were incubated for 1 h at room temperature. The sensors were then washed with deionized water and then rinsed by 5 s immersion in 37 °C PBST on a magnet stirrer at 400 rpm followed by drying with nitrogen. After the blocking step, the electrochemical signal of the functionalized sensor was measured using a potentiostat.

### 2.4. Characterization of Graphene Electrochemical Sensors

All electrochemical measurements were conducted using a portable potentiostat (Palmsens 4, PalmSens, Houten, The Netherlands). Characterization of the graphene electrode was carried out by cyclic voltammetry (CV) and electrochemical impedance spectroscopy (EIS). CV and EIS were utilized to characterize the electrode at each stage of functionalization and to evaluate the antibody–antigen interaction onto surfaces. All electrochemical measurements were performed in PBS solution, containing 2.5 mM Ferriferrocyanide and measured against the reference electrode. The CVs were performed from −1.2 to 1.1 V and a potential step of 0.01 V, with scan rates between 0.05 and 0.25 V/s. The EIS measurements were performed using a frequency range of 100 kHz to 0.1 Hz at 0.01 V AC voltage and a DC voltage of −0.31 V.

### 2.5. Spike and Virus Detection

For detection of the Spike protein, the sensors were incubated for 45 min 37 °C and 300 rpm shaking with 10 µL PBS (control), or 10 µL 260, 520, or 1040 nM of SARS-CoV-2 Spike S1-His protein (Sino biological, #40591-V08B1). Sensors were then washed with deionized water, rinsed by immersion in a 37 °C PBST (0.005% Tween-20) bath for 5 s, and dried under a gentle stream on nitrogen gas before performing electrochemical measurements. For detection of SARS-CoV-2 recombinant virus detection, a viral stock of 22E6 PFU/mL stored in Dulbecco’s Modified Eagle’s medium (DMEM) with 1% bovine serum albumin (BSA) and inactivated by dilution 1:1 in viral lysis buffer (100 mM Tris-HCl, 800 mM NaCl, pH 9.0, 1% Triton x-100, 1% BSA) was used. Before use on the immunosensor, an aliquot of viral stock and lysis buffer were purified using HiPPR™ detergent Removal Spin Columns (#88305, Thermo fisher) to remove the Triton-X detergent following the manufacturer’s manual. In short, the sample was diluted 1:3 with Milli-Q water and spun through the detergent removal resin for 2 min at 1500 g. PBS was used as a resin equilibrator, and the eluted volume was the same as input volume.

To electrochemically measure the spike protein, the electrochemical signal of the functionalized sensor was measured after the blocking step (background signal) and again after incubation with the analyte using square wave voltammetry (SWV) (analyte signal). SWV was conducted from −0.8 to 1.0 V using an amplitude of 0.25 V and a frequency of 10 Hz. To find the specific current change due to the spike binding to the sensor surface, the background signal was subtracted from the analyte signal using the PSTrace V5.8 software (PalmSens, Houten, The Netherlands).

### 2.6. Statistics

One-way ANOVA tests were carried out using Graphpad Prism 7. Where applicable, multiple comparisons were adjusted using Tukey’s test. SD values are calculated using the n-1 method as per standard convention.

## 3. Results and Discussion

### 3.1. Choice of Antigen and Validation of Antibody

The SARS-CoV-2 virus contains four major structural proteins, Spike, Nucleocapsid, Matrix, and Envelope, from which the Spike protein sequence varies most compared to its corresponding protein in SARS-CoV and MERS-CoV viruses. The choice of the Spike protein as antigen for the development of the biosensor was based on the potential to make a Sars-CoV-2 specific detection and its previously demonstrated high immunogenicity in a similar setting [23].

To validate specific binding between the Spike S1 protein and the monoclonal antibodies, an Enzyme-linked immunosorbent assay (ELISA) was performed first. Spike S1 protein was coated on the plates and the two anti-Sars-Cov2-Spike-S1 antibodies, and a secondary anti-human-igG HRP conjugated antibody was added subsequently. The results revealed that both antibodies bind to the antigen (Figure S1). To demonstrate the specificity of the antibodies 26 nM Lysozyme (370 ng/mL) and BSA (1730 ng/mL) were used as negative control antigens against the anti-spike S1 antibodies, and using either of them resulted in no detectable signal. The limit of detection of Sars-Cov-2 Spike S1 protein in the ELISA was determined to be approximately 3 ng/mL.

### 3.2. Functionalization and Characterization of Graphene Sensors for Electrochemical Detection of SARS-CoV-2

**Figure 1:**
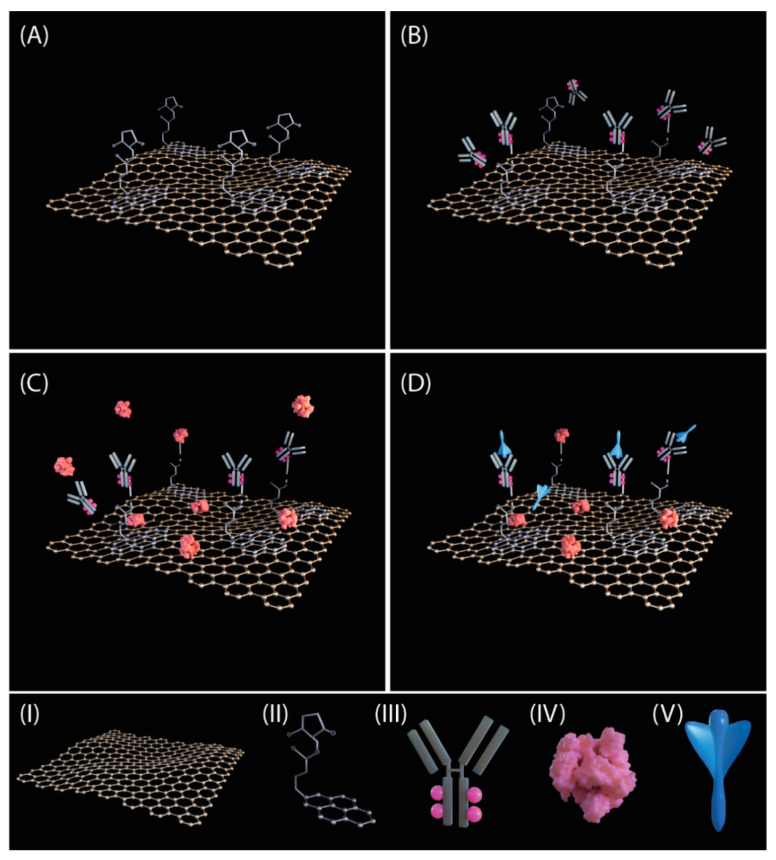
Schematics of functionalization and the concept of detection. (**A**) The graphene working electrode is functionalized with 1-pyrene butyric acid N-hydroxysuccinimide ester (PBASE) linker. (**B**) The spike-specific antibodies are immobilized to the electrode using the linkers. (**C**) Bovine Serum Albumin (BSA) is used to block free surface or linkers on the electrode. (**D**) Upon addition of the sample, only the spike protein will attach to the antibodies. (**I**) Graphene lattice; (**II**) 1-Pyrenebutyric acid N-hydroxysuccinimide ester (PBASE) linker; (**III**) spike-specific antibody; (**IV**) Bovine serum albumin (BSA) protein; (**V**) SARS-CoV-2 spike subunit 1 protein.

The sensor was produced by coating graphene working electrodes with a linker (Figure 1A) that can bind to a specific antibody against SARS-CoV-2 spike protein (Figure 1B). This approach is similar to the that one Seo et al. have used and characterized [23]. To block the excess reactive linkers remaining on the surface, the sensor is incubated with BSA. The sensor was then used to measure ferri/ferrocyanide, an electrochemically active chemical, after BSA blocking to obtain the background signal (Figure 1C). After analyte incubation, the spike protein should recognize and bind to the antibodies (Figure 1D).

**Figure 2:**
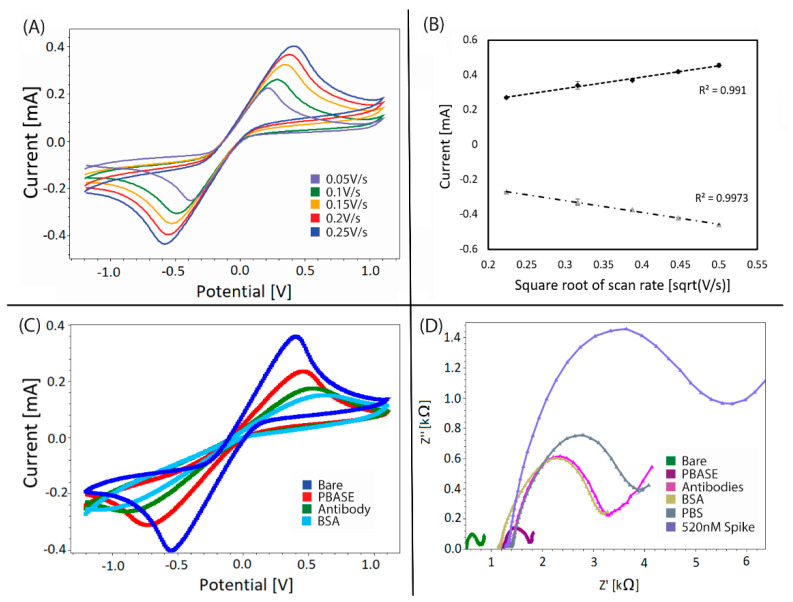
Diffusion-controlled electrochemical characterization (**A**) Cyclic voltammograms of the functionalized electrode as a function of the square root of scan rate, (**B**) Randles–Sevcik plot of peak current versus square root of scan rate. Electrochemical characterization of electrode functionalization. (**C**) Nyquist plot and (**D**) Cyclic voltammograms of each functionalization step.

The graphene sensor reversibility was evaluated by cyclic voltammograms using a 10 mM ferri/ferrocyanide solution in a 0.1 M KCl supporting electrolyte at scan rates from 50–250 mV/s (Figure 2A). Symmetrical voltammograms were observed. The half-wave potentials were evaluated for each voltammogram from the anodic and cathodic peak potentials, Epa and Epc, respectively, according to the standard equation (The standard equation: E0 = (Epa + Epc)/2), yielding a value of −91.5 ± 4.9 mV vs. the reference electrode. The numeric peak current ratio (Ipa/Ipc) was observed to be one for all voltammograms with a standard deviation of 0.015. The Randles–Sevcik plot demonstrates that Ipa and Ipc, respectively, are proportional to the square root of scan rates with R^2^ of 0.9919 and 0.9905 (Figure 2B). Although the mentioned characterization demonstrates characteristics of ideal reversible electron transfer and diffusion-controlled reaction, the peak potentials increase with increasing scan rates. This deems the sensor pseudo-reversible, which means that the sensor is sufficient for analytical determination of signal change in response to a given perturbation [27].

Electrochemical impedance spectroscopy and CV were performed to investigate the electrode behaviour after each surface modification step (Figure 2C,D). Cyclic voltammograms clearly show that the ferri/ferrocyanide electron transfer kinetics change from one step to another (Figure 2C). Ferri/ferrocyanide CV of the bare graphene electrode shows a well-defined characteristic reversible peak. The introduction of PBASE, antibodies and BSA on the surface leads to a decrease in electron-transport resistance with each step. Thus, the antibody immobilization and the blocking with BSA onto the activated electrode surface act as inert electron transfer relative blocking layers without completely hindering the diffusion of the ferri/ferrocyanide redox couple towards the electrode surface.

Electrochemical impedance spectroscopy was further performed to characterize the features of the different steps of the electrode modification (Figure 2D). The Nyquist plot of the different functionalization steps reveals changes in the electron-transfer resistance. Following the PBASE step, the electron transfer resistance slightly increased, which is also apparent from the cyclic voltammograms. The immobilization of antibodies on the electrode surface visibly increases the blocking of the electron transfer, demonstrating the formation of an insulating layer. The electron-transfer resistance remains constant upon blocking with BSA, indicating that most PBASE linkers were bound to antibodies. The subsequent addition of PBS to the sensor as a control analyte, resulted in a relatively small increase compared to the BSA-step, while the addition of 520 nM spike remarkably increased the electron-transfer resistance.

### 3.3. Detection of Spike Subunit 1 Using the Electrochemical Immunosensor

**Figure 3:**
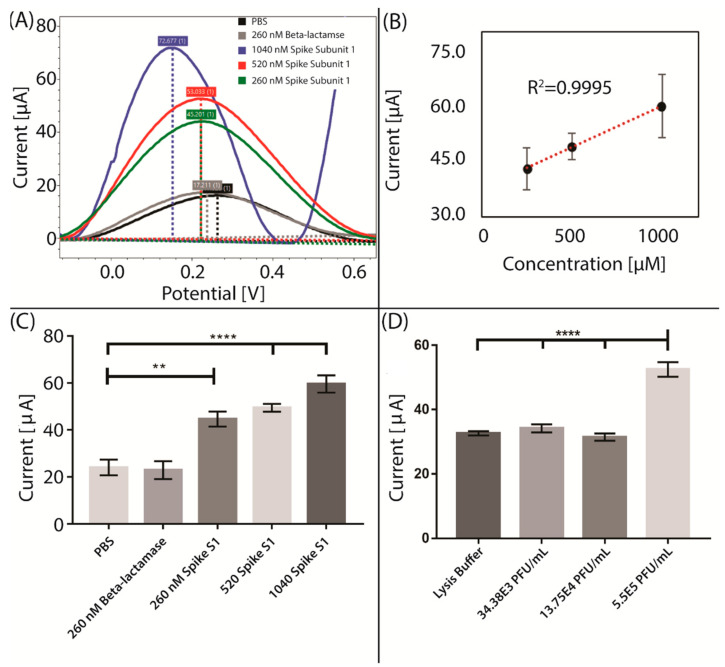
Current perturbation when measuring PBS, beta-lactamase and three different spike concentrations. (**A**) Square wave voltammograms of the signal difference of different spike concentrations and controls. (**B**) Linear regression between spike concentration and current perturbation. (**C**) Extracted peak height averages versus current of tested analytes. Bars are mean values from two separate experiments and batches of sensors with 3 sensors in each; with error bars showing SEM, *n* = 6. (**D**) Extracted peak height averages of tested analytes versus current when measuring viral lysis buffer and three different recombinant SARS-CoV-2 virus concentrations. Bars are mean values from 3 separate sensors, with error bars showing SEM, *n* = 3). Statistical significance was obtained using one-way ANOVA with adjusted *p*-values of *p* < 0.01 (**) and *p* < 0.0001 (****).

For quantitative analysis of Spike subunit 1 detection, Square Wave Voltammetry (SWV) was performed to detect the change of the ferri/ferrocyanide signal. The functionalized working electrode was incubated with dilutions of either Spike protein, PBS or beta-lactamase, as a control protein. Signal curves were obtained by scanning the potential from −0.8 to 1 V using SWV before and after incubation with the analyte. The analyte peak was then point-by-point subtracted from the background peak in the PSTrace software. The subtraction resulted in distinct peaks around 0.2 V. The resulting peak heights were used as a direct measure of the contribution of the analyte to total electron diffusion. Each analyte was measured six times, using three separate sensors in two separate experiments. The peak heights of each analyte were then averaged. Incubation with either PBS or the unspecific control protein beta-lactamase resulted in similar averaged peak heights at 24 and 22 µA, respectively. Three Spike S1 concentrations of 260 nM (20 µg/mL), 520 nM (40 µg/mL) and 1040 nM (80 µg/mL) all resulted in increased peak heights compared to the controls, at approximately 45, 49 and 60 µA, respectively (Figure 3A). The three spike S1 concentrations follow a linear correlation, with a regression coefficient of 0.9995 (Figure 3B). The data showed a statistically significant difference between the controls and all Spike concentrations (260 nM: *p* < 0.01; 520 nM and 1040 nM: *p* < 0.0001) (Figure 3C). Thus, the lowest concentration of Spike S1 protein detected using our proof-of-concept immunosensor was 260 nM (20 µg/mL).

### 3.4. Direct Detection of SARS-CoV-2 Using Electrochemical Immunosensor

To evaluate the ability of the novel proof-of-concept immunosensor for the electrochemical detection of SARS-CoV-2, we tested concentrations of recombinant virus ranging from 34.38 × 10^3^ to 5.50 × 10^5^ PFU/mL (Figure 3D). The virus stock was stored in Dulbecco’s Modified Eagle’s medium (DMEM) with 1% bovine serum albumin (BSA) and inactivated by dilution 1:1 in viral lysis buffer. The lysis buffer was tested on the sensor and was found to interfere with the signal until diluted to 2000× (data not shown). We, therefore, removed the Triton-X detergent from the solutions using a resin-based spin column before measuring on the inactivated virus and the lysis buffer control. Incubation with a purified and 20-times-diluted viral lysis buffer resulted in an average peak height of about 32.6 µA.

Three purified SARS-CoV-2 concentrations of 34.38 × 10^3^, 13.75 × 10^4^, and 5.50 × 10^5^ PFU/mL were measured and resulted in average peak heights of 34.1, 31.4, and 52.5 µA, respectively (Figure 3D). The signal from the highest concentration of 5.50E5 PFU/mL was statistically significant compared to the lysis buffer control (*p* < 0.0001). However, no difference in signal was detected for the two lower concentration samples, indicating that our detection limit is somewhere between 13.75 × 10^5^ and 5.5 × 10^5^ PFU/mL for our proof-of-concept immunosensor. Based on the linearity between the spike concentration and current perturbation seen in Figure 3B, the detected virus concentration corresponds to a spike concentration of ~690 nM. Thus, it is theoretically possible to reach lower virus detection limits without further modifications of the experimental setup. A Lancet study has measured the virus titer in nasopharyngeal swabs from two COVID-19-positive individuals, finding viral loads of 6.25 × 10^5^ and 3.0 × 10^7^ PFU/mL [28]. Thus, the viral concentrations detected by the immunosensor in this study are within the relevant physiological range.

To ensure that the sensor can be used to identify patients with mild SARS-CoV-2 infections, the limit of detection could be optimized further. One way to accomplish this would be to target the nucleocapsid protein instead of the spike protein. Preliminary data from our Simoa assay detected a considerably higher signal from the nucleocapsid protein than from the Spike protein (data not shown). Several commercially available antigen tests target the nucleocapsid protein with relatively high sensitivity [9]. However, as discussed earlier, the nucleocapsid protein shares high similarity to other coronaviruses, and thus could increase the risk of false-positive detection [10,11]. In future work, investigation of cross-specificity using other common human coronaviruses like HCoV-OC43, HCoV-HKU1, HCoV-229E, and HCoV-NL63 and rhinoviruses will give strong evidence for the risk of false-positive detection.

Additional characterization of the actual number of antibodies bound to the surface may shed light on whether a denser antibody immobilization can lead to higher sensitivity. Another way to enhance the spike specific signal from the current sensor functionalization setup could be a subsequent addition of redox-labelled nanoparticles functionalized with anti-spike antibodies on top of the bound spike proteins, thereby obtaining a specific redox signal.

### 3.5. Determination of Spike Protein Antigen in Recombinant SARS-CoV-2

Most antigen tests state a limit of detection in the weight or molar of the target analyte. However, limited information exists about the quantity of detectable antigen in SARS-CoV-2 samples, making it difficult to determine whether the detection limit is clinically relevant. This has prompted assay developers to investigate the correlation using their proof-of-concept assays [29]. We wanted to add to this discussion, and therefore investigated the amount of detectable full-length spike protein that was available for antibody binding in dilutions of recombinant SARS-CoV-2 virus. The virus titers are often given in plaque-forming units per millilitre (PFU/mL) which correlate to the amount of infectious viral particles. The single-molecule array (Simoa) assay was used to compare the signal from the available spike antigen in SARS-CoV-2 dilutions with known PFU/mL to known concentrations of recombinant spike subunit 1 protein. In short, the Simoa method uses the same reagents as conventional immunoassays such as ELISA but uses femtoliter-sized reaction chambers that are approximately 2 billion times smaller. This will result in a rapid buildup of fluorescence if a labelled protein is present on an antibody-labelled bead, which allows for single-molecule detection and, on average, offers 1000-fold greater sensitivity than conventional immunoassays [30,31]. The assay was able to detect recombinant Spike subunit 1 protein down to a concentration of approximately 0.5 pg/mL, and a SARS-CoV-2 viral titer at around 800 PFU/mL (Figure S2). The data revealed a linear correlation between the Spike subunit 1 protein concentration and the viral load of Triton-X inactivated SARS-CoV-2, as measured by the immunosensor. We found that using the Simoa assay, the signal from 1700 PFU of recombinant SARS-CoV-2 correlates to the signal from about 1 pg of pure Spike antigen. Using our proof-of-concept immunosensor we were able to detect Spike protein at a concentration of 20 ng/mL, while our lowest significant signal from SARS-CoV-2 was detected from 5.5 × 10^5^ PFU/mL. This correlates to 27.5 PFU of recombinant SARS-CoV-2 per pg of pure Spike antigen. The two assays are only an indirect comparison between the Spike concentrations in the pure form and from a viral solution, using only the signal size as a measure. The vastly different correlations show that they are highly assay dependent, likely attributable to the fundamentally different technologies used. Thus, we suggest that it is necessary to determine the correlation between the Spike protein and the viral load for every specific assay.

## 4. Conclusions

This work describes a proof-of-concept for a simple, label-free electrochemical immunosensor for the fast and direct detection of SARS-CoV-2 through the specific detection of the Spike subunit 1 protein, which is a unique biomarker for SARS-CoV-2. ELISA was used to validate the antibody–antigen interaction, while cyclic voltammetry and electrochemical impedance spectroscopy were used to characterize the individual layers of the electrode functionalization. It was possible to specifically detect Spike protein at 260 nM (20 µg/mL) while the signal from the control protein beta-lactamase at 260 nM (7.5 µg/mL) was equal to the background. The sensor was able to detect SARS-CoV-2 at a concentration of 5.5 × 10^5^ PFU/mL, which is within the physiologically relevant concentration range. Thus, we here present a novel proof-of-concept immunosensor able to detect SARS-CoV-2 in about 45 min, enabling portable on-site screening upon further optimizations of the detection limit. Additionally, we have demonstrated a correlation between the Spike concentration and the viral load using our sensor and a commercially available assay.

## Data Availability

Not applicable.

## Supplementary Materials

The following are available online at https://www.mdpi.com/1424-8220/21/2/390/s1, Figure S1: ELISA for validating the binding of anti-SARS-CoV-2 spike S1 antibodies to varying concentrations of Sars-Cov-2 Spike S1 protein. Figure S2: Viral load vs. spike subunit 1 concentration.
