# Supplementary material for: Rapid SARS-CoV-2 Detection Using Electrochemical Immunosensor
Source: Sensors (Basel). 2021 Jan 8;21(2):390. doi: 10.3390/s21020390 (PMC7827295; doi:10.3390/s21020390)
Supplement: Supplementary file 1 [file sensors-21-00390-s001.pdf]

Supplementary Information for Rapid SARS-CoV-2 detection using electrochemical immunosensor

Biljana Mojsoska[§,1] and Sylvester Larsen[§,1], Dorte Aalund Olsen[2,3], Jonna Skov Madsen[2,3], Ivan Brandslund[2,3], Fatima AlZahra'a Alatraktchi[*,1]

*Corresponding author, §Shared first authors

[1] Department of Science and Environment, Roskilde University, Universitetsvej 1, 4000 Roskilde, Denmark

[2] Department of Biochemistry and Immunology, Lillebaelt Hospital, University Hospital of Southern Denmark, Vejle, Denmark.

[3] Department of Regional Health Research, Faculty of Health Sciences, University of Southern Denmark, Odense, Denmark.

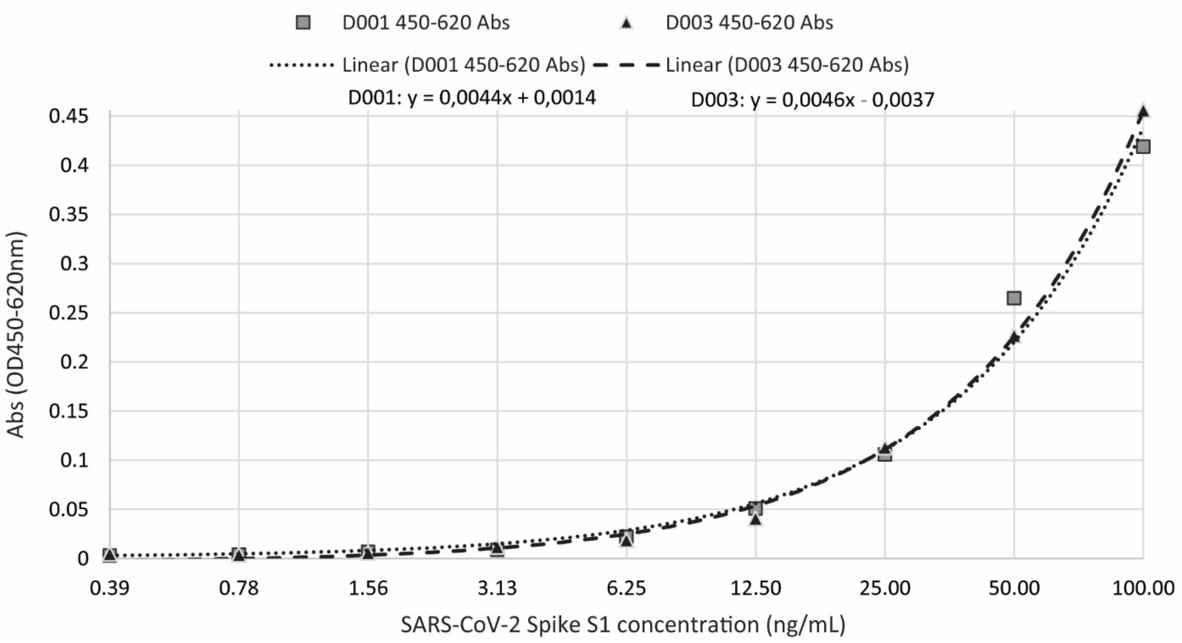

Figure S1 ELISA for validating the binding of anti-SARS-CoV-2 Spike S1 antibodies (#45150-D003 and #45150-D001) to varying concentrations of Sars-Cov-2 Spike S1 protein (#40591-V08B1).

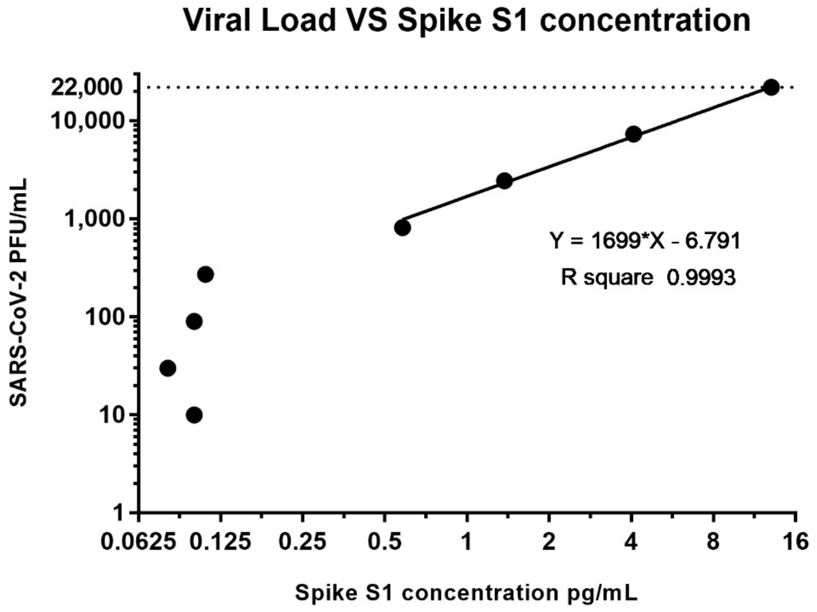

Figure S2 Comparison of Spike subunit 1 protein concentration and plaque-forming units of SARS-CoV-2 using the single-molecule array (Simoa™). Linear correlation was carried out using Graphpad Prism 7.
